# Society Meetings

**Published:** 1900-12

**Authors:** 


### SOCIETY MEETINGS.

The American Public Health Association at its recent meeting at Indianapolis voted to hold its next annual meeting at Buffalo. This was brought about largely through the influence of Drs. Ernest Wende and W. G. Bissell, who represented the Buffalo health department as delegates to the meeting.

The Medical Society of the State of New York will hold its ninety-fifth annual meeting at Albany, January 29, 30 and 31, 1901, under the presidency of Dr. A. M. Phelps, of 62 E. Thirty-fourth street, New York City. The business committee consists of Dr. Frank Van Fleet, 63 E. Seventy-ninth street, New York City, chairman; Dr. J. B. Ransom, Dannemora and Dr. F. H. Parker, of Auburn. Those who desire to read papers are requested to communicate promptly with one of the members of the business committee or the president on the subject.

As there will be a great many papers offered, and the time necessarily limited, it is suggested that papers be condensed as much as possible in reading, as they can be published more fully in the transactions. Arrangements for reduced fares can be made when purchasing railroad tickets.

---

THE Buffalo Academy of Medicine held meetings during the month of November, 1900, as follows:

*Section on Surgery.*—Monday evening, November 5th. Program: Differential diagnosis in diseases of the gall bladder, George Emerson Brewer, Surgeon Roosevelt Hospital, New York City; discussion opened by Roswell Park.

*Section on Medicine.*—Tuesday evening, November 13th. Program: A review of the modern surgical and medical treatment of epilepsy, L. Pierce Clark, physician Craig Colony for Epileptics, Sonyea, N. Y.; Paresis and cerebral syphilis, Arthur W. Hurd; discussion by Roswell Park and James N. Putnam. A case of asthenic bulbular paralysis, William C. Krauss. Report of a case of pseudo-hypertrophic muscular paralysis, Fridolin M. Thoma; discussion by Dewitt H. Sherman.

*Section on Ophthalmology, Otology, Rhinology and Laryngology.*—Monday evening, November 19th. Program: Exhibition of a case of total removal of thyroid gland, G. F. Cott. Discussion: The relation of tuberculosis of the larynx to pulmonary tuberculosis; discussion opened by De Lancey Rochester, followed by E. L. Shurly, of Detroit, Mich., and John H. Pryor.

*Section on Pathology.*—Tuesday evening, November 20th. Program: Pathology of stone in bladder, W. D. Green. Pathology of arthritis deformans, and exhibition of cases, Herman Mynter.

*Section on Obstetrics.*—Tuesday evening, November 27th. Subject to be announced, C. C. Frederick.

---

THE Niagara Falls Academy of Medicine held its regular monthly meeting at the office of Dr. W. H. Potter, 17 Sugar Street, Echota, on Monday evening, November 12, 1900, at 8.45 p.m. Papers were read by Dr. A. L. Chapin and Dr. O. E. McCarty.

The St. Louis Academy of Medical and Surgical Sciences elected the following officers for 1901, at its last regular meeting: President, Emory Lamphear; senior vice-president, Carl Pesold; junior vice-president, H. S. P. Lure; secretary, O. L. Suggett; treasurer, G. M. Phillips; orator, Wm. Porter; librarian, H. G. Nicks.

---

The Southern Surgical and Gynecological Association held another of its famous annual meetings at Atlanta, Ga., November 13, 14 and 15, 1900. The association was presided over by Dr. A. M. Cartledge, of Louisville, Ky., who made an ideal president in appearance, manner and method, and his presidential address was replete in important surgical truths faultlessly delivered.

In attendance were representative men from all sections of the country, many of whom read papers of instructive interest which were discussed, for the most part, with incisive clearness and detail.

The leading members of the medical profession of Atlanta provided elaborate and delightful entertainments for the visitors, among which were a smoker on Tuesday evening and a reception on Wednesday evening, both given at the Kimball House. Besides these there were private dinners and luncheons given by Drs. Westmoreland, Noble, McRae, Holmes, Elkin and Cooper.

Dr. M. H. Simmons, of Charleston, S. C., was elected president in succession, and the next meeting will be held at Richmond, Va., beginning on the second Tuesday of November, 1901, at which Dr. George Ben Johnston will serve as chairman of the committee of arrangements.

Dr. W. E. B. Davis, of Birmingham, Ala., who has been secretary of the association from its foundation, voluntarily retired from the position and his mantle falls upon Dr. W. D. Haggard, Jr., of Nashville, one of the active younger members of the association. In surrendering his office Dr. Davis leaves in the hands of his successor a splendidly organised body of professional workers, that owes much to his faithfulness and great executive capability. The medical profession of the South owes more to Dr. Davis than it can repay in many a year, no matter how highly it may choose to honor him.

